# PD05 - Asthma and allergy from infancy into school age – the allergic march revisited

**DOI:** 10.1186/2045-7022-4-S1-P5

**Published:** 2014-02-28

**Authors:** Emma Goksör, Bernt Alm, Rolf Pettersson, Per Möllborg, Laslo Erdes, Petra Loid, Nils Åberg, Göran Wennergren

**Affiliations:** 1 Department of Pediatrics, University of Gothenburg, Gothenburg, Sweden

## Background

The allergic march describes a proposed natural course of allergic disease during childhood. However, different patterns of allergic morbidity have been suggested. The aim of this study was to describe the prevalence of doctor-diagnosed asthma and allergic manifestations from infancy into school age and the relationship between early manifestations and the prevalence of asthma at school age.

## Methods

Data were obtained from a prospective, longitudinal study of a cohort of children born in western Sweden. The parents answered questionnaires at 6 months and 1, 4.5 and 8 years of age. The response rate at 8 years was 80% of the questionnaires distributed (4,051/5,044), that is 71% of the families entering the study (4,051/5,654).

## Results

The prevalence of recurrent wheeze decreased from infancy to school age (5,4% to 3,4%), but the prevalence of doctor-diagnosed asthma increased from 2,1% in infancy to 5,7% at 8 years of age. The prevalence of doctor-diagnosed eczema was more than halved from infancy to school age (20.9%, 8.6 % and 7.9% at age 1, 4 and 8 years, respectively), while doctor-diagnosed food allergy decreased slightly (4.9%, 2.8% and 3.5%). Doctor-diagnosed rhinitis increased from 1.7 % at age 4 to 5.6 % at age 8 years.

The prevalence of school age asthma increased with the number of allergic manifestations that was seen during infancy. Of those with 3 early manifestations (eczema, food allergy and wheeze treated with inhaled corticosteroids) more than 60% had doctor-diagnosed asthma at 8 years, compared with only 3% school age asthma among those who were symptom-free in infancy.

## Conclusion

Doctor-diagnosed asthma and rhinitis increase, while eczema and food allergy decrease from infancy into school age in line with the proposed allergic march. In children with several early allergic manifestations in infancy two out of three have doctor-diagnosed asthma at age 8.

**Figure 1:**
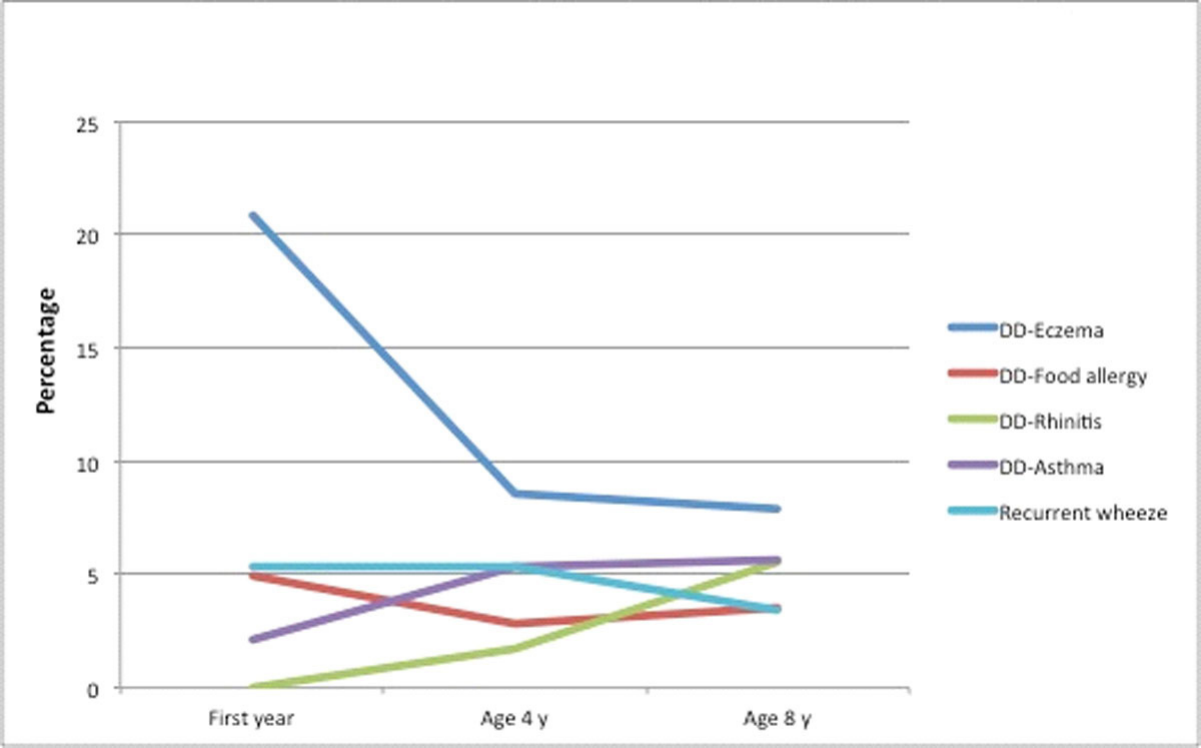
Prevalence of doctor-diagnosed allergic manifestations, doctor-diagnosed asthma and recurrent wheeze during childhood in the cohort

**Figure 2:**
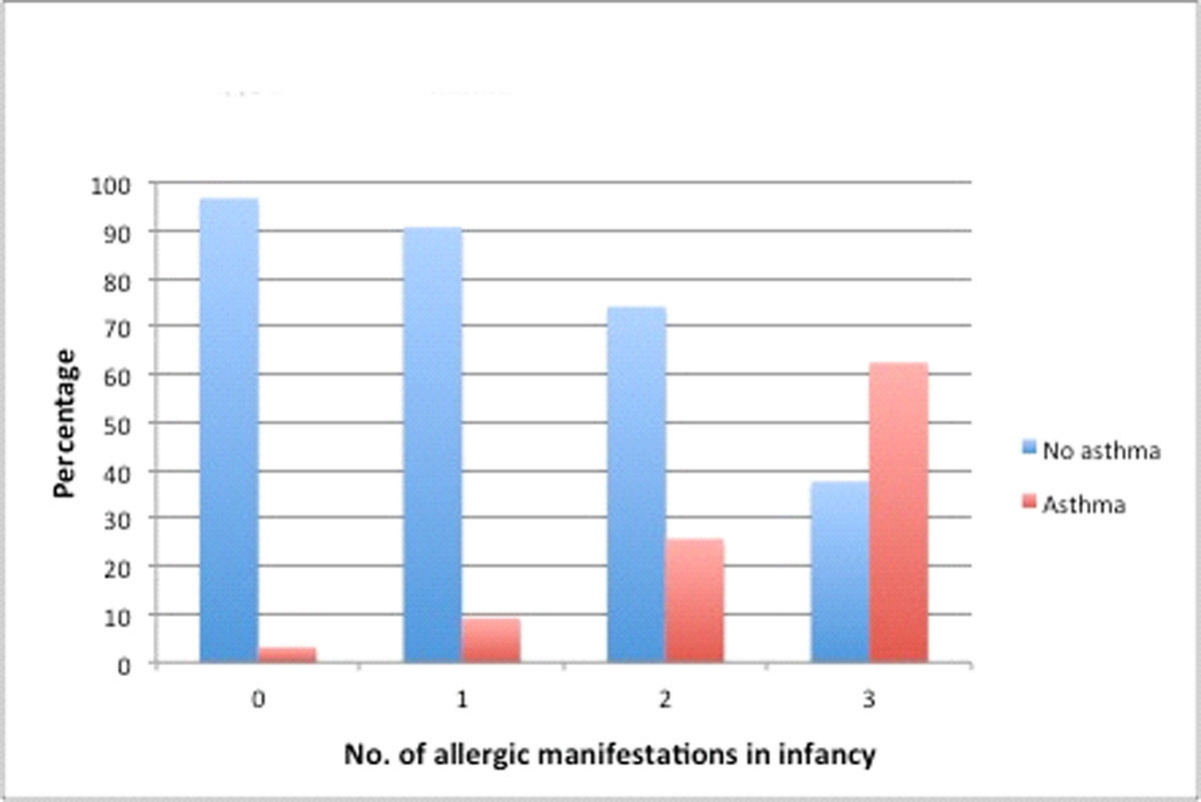
Prevalence of doctor-diagnosed asthma at age 8 years among subjects with 0 to 3 manifestations, i.e. doctor-diagnosed eczema, food allergy or ICS-treated-wheeze in infancy

